# 

**Published:** 1870-10

**Authors:** 


### ASSOCIATION.

The annual meeting of the Ohio State Dental Society will be held Tuesday, the 6th of December next, at Columbus, and will, by resolution of the last meeting, continue three days. The indications now are that it will be by far the largest meeting of this body ever held. It is earnestly expected and desired that all the earnest workers and well-wishers of the profession in the State will be present. There will be much important business before the meeting. There is much preparation making for the introduction of practical subjects, and judging from the plan proposed, we doubt not they will be better elaborated than ever before. Then there are other subjects of vital importance that will claim attention and must be considered at that meeting. There will be two members of the State Board of Examiners to elect. There will be matters brought before the meeting of special interest to all the profession in the State ; and brothers, if you wish them properly adjusted, be on hand. Let every one come prepared to do his part. T.

Dr. Feemster, of Greencastle, Ind., has so far perfected his method of casting aluminum plates for artificial teeth that he is ready to introduce it to the profession for general use. By it we know he is able to accomplish all he claims. He makes very beautiful plates, and very perfect ones, too. The casting Dental plates of aluminum is a matter that has been long sought for. Knowing the value of the metal in many respects for the purpose, quite a number of the profession have experimented with it from time to time. Two or three others at about the same time with Dr. Feemster succeeded in finding processes by which they can cast plates; none, we think, however, with better success or greater facility than Dr. Feemster.

This is certainly a great point gained, and every such effort should receive the hearty support and co-operation of every member of the profession. Dr. Feemster will himself be among the profession very soon. T.

AMERICAN DENTAL ASSOCIATION—Meets on the first Tuesday in August next year at Greenbrier, White Sulphur Springs, Va. *Pres.* W. H. MORGAN, Nashville, Tenn. *Rec. Sec.* M. S. DEAN, Chicago.

AMERICAN DENTAL CONVENTION.—Meets in New York, Sept. 1870 *Pres.* Dr. J. G. AMBLER. *Sec.* and *Treas.* Dr. J. H. SMITH, New Haven, Conn.

*List of Societies represented in the American Dental Association.*

AMERICAN ACADEMY OF DENTAL SCIENCE—Meets at Boston, Mass. *Pres.* DANIEL HARWOOD, M. D., of Boston. *Sec.* E. N HARRIS, D. D. S., of Boston.

BROOKLYN DENTAL SOCIETY—Meets semi-monthly. *Pres.* C. D. Cook. *Rec. Sec.* E L. CHILDS. *Cor. Sec.* WM. JARVIE, Jr.

BUCKS COUNTY DENTAL ASSOCIATION, (PA.) Meets semi-annually on the first Monday in May and November. Next meeting at Newton, in November. *Pres.* H. P. Yerkes, *Sec.* G. W. Adams.

BUFFALO DENTAL SOCIETY—Meets monthly at Buffalo. *Pres.* B T. Whitney, *Rec. Sec.* S. A. FREEMAN.

BOSTON DENTAL INSTITUTE. Meets first Tuesday of each month. *Pres.* D. G. Williams. *Cor. Sec.* D. S. Dickerman.

CUMBERLAND VALLEY DENTAL SOCIETY—Meets semi-annually.* *Pres.* J. L. SUESSEROTT, of Chambersburg. *Sec.* GEO. W. NEIDICH, of Carlisle, Pa.

CINCINNATI DENTAL SOCIETY—Meets semi-monthly at Cincinnati. *Pres.* Geo. WATT, *Rec. Sec.* Wm. TAFT.

CENTRAL OHIO DENTAL ASSOCIATION—Meets Annually on the second Tuesday of July. *Pres.* S. P. HARRINGTON, Newark. *Sec.* G. W. DECAMP Mansfield, O.

CHICAGO DENTAL SOCIETY—Meets monthly at Chicago. *Pres.* GEO. H. CUSHING. *Rec. and Cor. Sec.* EDGAR D. SWAIN.

CONNECTICUT VALLEY DENTAL ASSOCIATION—Meets semi-annually, second Tuesday of May and October, *Pres.* A. A. HOWLAND, Barre, Vt. *Rec. Sec.* W. H. JONES, of Boston, Mass.

CONNECTICUT STATE DENTAL ASSOCIATION—*Pres.* H. L. SAGE, Bridgeport. *Cor. Sec.* JOHN CODY, Hartford. *Rec. Sec.* C. A. POWERS, Hartford.

CHARLESTON DENTAL ASSOCIATION.*—*Prest.* I. B. PATRICK. *Sec. and Treas.* T. F. CHUPEIN.

DELAWARE DENTAL ASSOCIATION—Meets semi-annually. *Pres.* E, W. HAINES, Newark. *Sec.* C. R. JEFFRIES, Wilmington.

EAST TENNESSEE DENTAL ASSOCIATION—Meets annually at Knoxville. Tenn. *Pres.* J. A. CRAZIER. of Knoxville *Rec. Sec.* W. F. FOWLER, Tenn.

FOREST CITY SOCIETY OF DENTAL SURGEONS. *Pres.* B. Strickland, Cleveland. *Rec. Sec.*, H. L. Ambler, Cleveland. *Cor. Sec.*, Chas. Buffett, Cleveland.

GEORGIA STATE DENTAL SOCIETY—Next meeting at Atlanta, July 30th, 1870. *Pres.* F. Y. CLARK; *Cor. Sec.* J. P. H. BROWN, Augusta.

HARRIS DENTAL ASSOCIATION OF LANCASTER, Pa—Next meeting at Columbia, on Thursday the [illegible]th of Nov. next. *President*, SAM'L. WELCHENS. *Sec.* WM. NICHOLS AMER.

HUDSON RIVER ASSOCIATION OF DENTAL SURGEONS—Meets on the second. Wednesdays of May, September and January. *Pres.* L. S. STRAW, Newburg N.Y. *Rec. Sec.* T. W. DuBOIS, Pougkeepsie, N. Y.

HUDSON VALLEY DENTAL ASSOCIATION—meets monthly at Troy, N. Y. *Pres.* L. C. WHEELER, *Rec. Sec.* S. G. ANDRES, *Cor. Sec.* S. D. FRENCH.

LLINOIS STATE DENTAL SOCIETY—Meets annually. *Pres.* M S. Dean, of Chicago. *Sec* C. S. SMITH, of Springfield.

INDIANA STATE DENTAL SOCIETY—Meets annually at Indianapolis on the last Tuesday of June. *Pres.* W. C. STANLEY, of Dublin. *Rec. and Cor. Sec.* S. B. Brown.

IOWA STATE DENTAL SOCIETY—Meets annually. *Next meeting at Iowa City, on the second Tuesday of July. *Pres.* J. HARDMAN, of Muscatine. *Rec. Sec.* A. B. MASON, of Waterloo. *Cor. Sec.* J. P. WILSON, of Burlington.

LAKE ERIE DENTAL ASSOCIATION.—Meets Annually.* *Pres.* B.T. WHITNEY, Buffalo N. Y., *Sec.* W. E. MAGILL, Erie, Pa.

LEBANON VALLEY DENTAL ASSOCIATION—*Prest.* W. K. BRENIZER, *Sec.* S. H. GUILFORD.

MAD RIVER VALLEY DENTAL SOCIETY—Meets Semi annually on the first Tuesday of May next. Next meeting at Cincinnati, O. *Pres.* J. H. PAINE, Middletown, O. *Sec.*, S. B. TIZZARD, Troy, O.

MAINE DENTAL SOCIETY—Meets in August, prox., at Biddeford. *Pres.* THOS. HALEY. *Sec.* E. G. ROBERTS.

MARYLAND ASSOCIATION OF DENTISTS—Meets the last Thursday of every month. *Pres.*, J. B. BEAN, *Sec.*, S. M. FIELD.

MASSACHUSETTS DENTAL SOCIETY—Meets monthly. *Pres.*, T. H. Chandler, *Rec. Sec.* A. Brown, *Cor. Sec.* E. Blake.

MICHIGAN DENTAL ASOCIATION—Meets annually second Tuesday of Oct. at Jackson *Pres.* E. S. HOLMES, of Grand Rapids; *Rec. and Cor. Sec.* G. H. MOSHER, of Jackson.

MUSKINGUM VALLEY DENTAL ASSOCIATION—Meets on first Monday of each month at Zanesville, O. *Pres.* W. M. Herriot, of Zanesville, O., *Sec.* W. W. Chapelea Zanesville, O.

MISSISSIPPI VALLEY DENTAL ASSOCIATION—Meets annually at Cincinnati, on the first Wednesday in March. *Pres.* W. H. MORGAN, Nashville. *Rec. Sec.* C. M. WRIGHT, Cincinnati, O. *Cor. Sec.* N. W. Williams, Xenia, O.

MISSOURI DENTAL ASSOCIATION—Meets on the first Tuesday of June, in St. Louis. *Pres.* H. JUDD, St. Louis, *Sec.* W. H. EAMES, St. Louis.

MISSOURI VALLEY DENTAL SOCIETY.* Meets on the second Tuesday in July and January. *Pres.* E. J. WOODBURY of Council Bluffs, Iowa. *Sec. and Treas.* J. F, Sanborn, Tabor, Iowa.

MERRIMAC VALLEY DENTAL SOCIETY—Meets semi annually first Tuesday of Oct and May.* *Pres.* I. H. KIDDER, Mass. *Rec. Sec.* A. M. DUDLEY, of Lowell. *Cor. Sec.* D. T. PORTER, Lawrence, Mass.

MEMPHIS DENTAL SOCIETY.—Meets on the first Tuesday of each month. *Pres* W. T. Arrington, *Sec.* V. F. Elliott. *Treas.* C. L. Harris.

NASHVILLE DENTAL ASSOCIATION—Meets on the second Tuesday of each month *Pres.* J. C. Ross, *Sec.* R. R. FREEMAN, *Cor. Sec.* S. J. COBB.

NEW YORK STATE DENTAL SOCIETY—Meets at Albany on the last Tuesday of June next. *Pres.* L. W. ROGERS, of Utica. *Sec.* CHAS. BARNS, of Syracuse *Cor. Sec.* W. H. ATKINSON, New York. *State Censors*, S. H. McCALL, Binghamton, 6th Dist. R. G. SNOW, Buffalo, 8th Dist.

NEW HAVEN DENTAL SOCIETY—Meets on the first Wednesday of each month at New Haven. *Pres.* J. T. METCALF, *Rec. and Cor. Sec.* C. L. SMITH, of New Haven.

NEW LONDON COUNTY DENTAL SOCIETY—Meets monthly.* *Pres.* R. W. BROWNE. *Rec. Sec.* WM. ALLENDER

NORTHERN OHIO DENTAL ASSOCIATION—Meets Semi-annually, (first Tuesday of May and October,) in Cleveland. *Pres.* W. P. HORTON, *Rec. Sec.* H L. AMBLER, *Cor. Sec.* CHAS. BUFFETT, Cleveland.

NORTHERN IOWA DENTAL ASSOCIATION—Meets annually, on the 2d Tuesday in June, 1870. Next meeting at Waterloo. *Pres.* E. S. CLARK, Dubuque. *Rec. Sec.* J. Z. ABBOTT, Manchester. *Cor. Sec.* A. B. MASON, Waterloo.

OHIO DENTAL COLLEGE ASSOCIATION — Meets annually in March, at Cincinnati *Pres.* Geo. H. Cushing, Chicago, Ill. *Rec Sec.* J. TAFT, of Cincinnati.

OHIO STATE DENTAL ASSOCIATION.—Meets semi-annually, at Columbus. [Nex meeting—first Tuesday of December.] *Pres.* F. H. REHWINKLE, Chillicothe, *Rec. Sec.* Wm. M. HERRIOTT, Zanesville, O. *Cor. Sec.* A. W. MAXWELL, Galion, O.

ODONTOGRAPHIC SOCIETY OF PENNSYLVANIA—Meets monthly in Philadelphia Next meeting, Wednesday, Sept. 1st. *Pres.* J. H. McQUILLEN. *Rec. Sec.* A. BOICE *Cor. Sec.* T. C. STELLWAGEN.

ODONTOLOGICAL SOCIETY OF NEW YORK CITY—Meets the second Tuesday of each month.* *Pres.* C. E. FRANCIS. *Rec Sec.* C. F. IVES.

OLD COLONY DENTAL ASSOCIATION — Meets quarterly.* *Pres.* C. G. Davis *Rec. Sec.* L. W. PUFFER, North Bridgewater, Mass. *Cor. Sec.* N. C. FOWLER, Yar mouth, Mass.

PENNSYLVANIA ASSOCIATION OF DENTAL SURGEONS—Meets monthly in Ph *Pres.* E. WILLIAMS. *Rec. Sec* JAMES TRUMAN *Cor. Sec.* E. T. DARBY.

PITTSBURG DENTAL ASSOCIATION—Meets first Tuesday of each month in Pittsbu *Pres.* C. L. WESTENBURG. *Rec. Sec.* J. D. WHITE.

SAN FRANCISCO DENTAL ASSOCIATION.—Meets monthly. *Pres.* HENRY AUSTIN *Cor. Sec.* H. E. KNOX *Rec. Sec.* A. B. WOOD.

POUGHKEEPSIE DENTAL SOCIETY.—Meets monthly, *Pres.* J. H. Mann, *Sec.* H. F. Clark.

ST. LOUIS DENTAL SOCIETY—Meets monthly. *Pres.* ISAAC COMSTOCK. *Sec.* R. J. POORE

SUSQUEHANNA DENTAL ASSOCIATION—Meets semi-annually*. *Pres.* C. S. BECK M. D. *Rec. Sec.* R. C. BURLEM.

SOCIETY OF DENTAL SURGEONS OF THE CITY OF NEW YORK—Meets semi monthly in New York. *Pres.* B. W FRANKLIN. *Rec. Sec.* J. S. LATIMER. *Cor. Sec* T. H. BURRAS.

SOUTEHRN DENTAL ASSOCIATION.—Meets annually.* Next meeting the 2d Wednesday in April, 1870, at Charleston, S. C. *Pres.* JAS. KNAPP. *Rec. Sec.* Prof. J. G. ANGELL, New Orleans

STATE DENTAL SOCIETY OF PENNSYLVANIA.—Meets annually Second Tuesday in June.* Next meeting in Gettysburg, 1871. *Pres.* JOHN McCALLA, Lancaster. *Rec. Sec.* C. H. BAGLEY, Meadville. *Cor. Sec.* SAMUEL WELCHENS, Lancaster.

TENNESSEE DENTAL ASSOCIATION.—Meets semi-annually. *Pres.* W. T. Arrington of Memphis, *Sec.* —— of Somerville.

TUSCARAWAS VALLEY DENTAL SOCIETY.—The annual meeting in Nov. 1870. *Pres.* JOHN McKINLY, Uhricsville. *Sec.* L. E. DISNEY, Coshocton.

WASHINGTON CITY DENTAL ASSOCIATION—Meets on the first Monday of each month. *Pres.* R. F. HUNT. *Sec.* J. C. SMITH. *Librarian*, H. N. WADSWORTH.

**Place of meeting fixed by appointment*

### DISTRICT DENTAL SOCIETIES OF THE STATE OF NEW YORK.

*1ST DISTRICT DENTAL SOCIETY.—*Pres.* A. L. Northrop, New York City. *Sec.* O. A. Jarvis, New York City.

2D DISTRICT DENTAL SOCIETY.—*Pres.* W. B. Hurd, Brooklyn. *Rec. Sec.* Wm. Jarvie Jr., Brooklyn. *Cor. Sec.* L S. Straw, Newburg.

3D DISTRICT DENTAL SOCIETY.—Meets at Albany semi-annually and annually. *Pres* J. A. Perkins, Troy. *Sec.* S. J. Andress, of Albany.

*4TH DISTRICT DENTAL SOCIETY.—*Pres.* F. C. Rich, Saratogo Springs. *Sec.* C. A Elmore, Fort Edwards.

*5TH DISTRICT DENTAL SOCIETY.—*Pres.* G. A. Foster, Utica *Sec.* Charles Barnes, Syracuse.

*6TH DISTRICT DENTAL SOCIETY.—*Pres.* A. M. Holmes. *Sec.* C. A. Perkins.

*7TH DISTRICT DENTAL SOCIETY.—*Pres.* A. G. Coleman, Canandagua. *Sec.* H. S. Miller, Rochester.

8TH DISTRICT DENTAL SOCIETY.—Meets annually on the 1st Tuesday in May, at Buffalo. *Pres.* L. W. Bristol, Lockport. *Sec.* S. A. Freeman, Buffalo.

THE 7TH AND 8TH DISTRICT SOCIETIES—Hold a union meeting on the first Tuesday of October, alternately at Buffalo and Rochester.

* Place and time of meeting fixed by appointment.

### EXCHANGES,

ST. LOUIS MEDICAL AND SURGICAL JOURNAL,
BOSTON MEDICAL AND SURGICAL JOURNAL,
THE PACIFIC MEDICAL AND SURGICAL JOURNAL, SAN FRANCISCO,
BUFFALO MEDICAL AND SURGICAL JOURNAL,
PHILADELPHIA MEDICAL AND SURGICAL REPORTER,
CINCINNATI LANCET AND OBSERVER,
DENTAL COSMOS, PHILADELPHIA.
L'ART DENTAIRE, PARIS, FRANCE.
BRAITHWAITE'S RETROSPECT, N. Y.
THE DENTAL TIMES, PHILADELPHIA,
LADIES' REPOSITORY, CINCINNATI.
HALL'S JOURNAL OF HEALTH, N. Y,
CHICAGO MEDICAL EXAMINER.
THE DETROIT REVIEW OF MEDICINE AND PHARMACY.
MEDICAL RECORD, N Y.
NASHVILLE JOURNAL OF MEDICINE AND SURGERY,
AMERICAN ECLECTIC MEDICAL REVIEW, N. Y.
AMERICAN JOURNAL OF DENTAL SCIENCE, BALTIMORE.
THE UNITED STATES MEDICAL AND SURGICAL JOURNAL. CHICAGO,
NEW ORLEANS JOURNAL OF MEDICINE.
WESTERN JOURNAL OF MEDICINE. INDIANAPOLIS, IND.
AMERICAN AGRICULTURIST, N. Y,
DENTAL OFFICE AND LABORATORY, PHILA.
BOSTON JOURNAL OF CHEMISTRY.
CINCINNATI MEDICAL REPERTORY.
CHICAGO MEDICAL INVESTIGATOR.
JOURNAL OF APPLIED CHEMISTRY, N. Y.
DRUGGISTS' CIRCULAR AND CHEMICAL GAZETTE, NEW YORK.
DER ZAHNARZT, LEIPZIG.
DEUTSCHE VIERTELJAHRSSCHRIFT, NUREMBURG.
CANADA JOURNAL OF DENTAL SCIENCE, MONTREAL.
OHIO CONVENTION REPORTER, COLUMBUS.
INDIANA JOURNAL OF MEDICINE.
JOURNAL OF THE GYNÆCOLOGICAL SOCIETY, BOSTON.
MEDICAL AND SURGICAL REPORTER, SALEM, OREGON.
THE TECHNOLOGIST, NEW YORK.
WOOD'S HOUSEHOLD MAGAZINE, NEWBURG, N. Y.
POPULAR SCIENCE REVIEW, LONDON, ENGLAND.
MISSOURI DENTAL JOURNAL, ST. LOUIS.
MEDICAL BULLETIN, BALTIMORE, MD.
ECLECTIC MEDICAL JOURNAL, CINCINNATI.

### The Dental Register

### OF THE WEST.

*Issued Monthly, at $3 a Year in Advance.*

DEVOTED TO THE INTERESTS OF THE DENTAL PROFESSION.

EDITED BY J. TAFT AND G. WATT.

The volume commences in January and closes in December.

The Register being issued monthly is always fresh, therefore indispensable to the practitioner who desires to keep posted up with the new inventions and improvements which are daily developed. Neither expense nor effort will be spared to give value and variety to its contents. Articles will be illustrated with well executed engravings whenever they will add to the interest or assist in explanation.

Its extensive circulation and frequency of issue makes it one of the BEST DENTAL ADVERTISING MEDIUMS in the United States.

RATES OF ADVERTISING.

One page, 1 year, $50. Half page, 1 year, $30. Quarter page, or card, 1 year $20.

All advertising bills payable in advance, or at furthest after the issue of the first number containing the advertisement. All transient advertisements to be paid for in advance.

☞ CONTRIBUTIONS SOLICITED.

Address, J. TAFT, or "DENTAL REGISTER," Cincinnati Ohio.

Business Communications to

J. TAFT, Publisher,

No. 117 West Fourth St.

